# A *de novo EGR2* variant, c.1232A > G p.Asp411Gly, causes severe early-onset Charcot-Marie-Tooth Neuropathy Type 3 (Dejerine-Sottas Neuropathy)

**DOI:** 10.1038/s41598-019-55875-4

**Published:** 2019-12-18

**Authors:** Bianca R. Grosz, Natasha B. Golovchenko, Melina Ellis, Kishore Kumar, Garth A. Nicholson, Anthony Antonellis, Marina L. Kennerson

**Affiliations:** 1 Northcott Neuroscience Laboratory, ANZAC Research Institute, Concord, NSW Australia; 2 Sydney Medical School, University of Sydney, Sydney, NSW Australia; 3 Department of Human Genetics, University of Michigan Medical School, Ann Arbor, MI USA; 4 Molecular Medicine Laboratory, Concord Repatriation General Hospital, Concord, NSW Australia; 5 Department of Neurology, Concord Repatriation General Hospital, Concord, NSW Australia; 6 Department of Neurology, University of Michigan Medical School, Ann Arbor, MI USA

**Keywords:** Medical genetics, Mutation, Peripheral nervous system

## Abstract

EGR2 (early growth response 2) is a crucial transcription factor for the myelination of the peripheral nervous system. Mutations in *EGR2* are reported to cause a heterogenous spectrum of peripheral neuropathy with wide variation in both severity and age of onset, including demyelinating and axonal forms of Charcot-Marie Tooth (CMT) neuropathy, Dejerine-Sottas neuropathy (DSN/CMT3), and congenital hypomyelinating neuropathy (CHN/CMT4E). Here we report a sporadic *de novo EGR2* variant, c.1232A > G (NM_000399.5), causing a missense p.Asp411Gly substitution and discovered through whole-exome sequencing (WES) of the proband. The resultant phenotype is severe demyelinating DSN with onset at two years of age, confirmed through nerve biopsy and electrophysiological examination. *In silico* analyses showed that the Asp411 residue is evolutionarily conserved, and the p.Asp411Gly variant was predicted to be deleterious by multiple *in silico* analyses. A luciferase-based reporter assay confirmed the reduced ability of p.Asp411Gly EGR2 to activate a *PMP22* (peripheral myelin protein 22) enhancer element compared to wild-type EGR2. This study adds further support to the heterogeneity of EGR2-related peripheral neuropathies and provides strong functional evidence for the pathogenicity of the p.Asp411Gly *EGR2* variant.

## Introduction

Charcot-Marie-Tooth (CMT) neuropathy is group of degenerative motor and sensory peripheral neuropathies which are clinically and genetically heterogenous. Pathogenic variants in over 90 genes cause CMT, and whole-exome sequencing (WES) is now an effective tool for screening known causative genes in unsolved CMT families. Pathogenic variants in the *EGR2* gene (early growth response 2) cause a broad spectrum of peripheral neuropathy phenotypes. This includes two forms of severe early-onset peripheral neuropathy, Dejerine-Sottas neuropathy (DSN/CMT3)^1–3^ and congenital hypomyelinating neuropathy (CHN/CMT4E)^4,5^, as well as adult-onset demyelinating CMT1D with mild-moderate symptoms^3–12^ and variable-onset axonal CMT with varied symptom severity^13,14^.

*EGR2* encodes a C_2_H_2_-type zinc-finger transcription factor that regulates the expression of genes involved in the formation and maintenance of myelin, including *GJB1* (gap junction beta 1), *MPZ* (myelin protein zero), *MBP* (myelin basic protein), *MAG* (myelin associated glycoprotein), *PRX* (periaxin), and *PMP22* (peripheral myelin protein 22)^4,15–18^. Approximately half of the reported disease-associated *EGR2* variants are *de novo* and are associated with a severe phenotype^13^. Here, we report a sporadic *de novo EGR2* variant in the third zinc-finger domain, c.1232A > G p.Asp411Gly, discovered through WES candidate gene screening. This variant manifested as a severe DSN phenotype with early onset at two years of age.

## Results

### Clinical history and neurological examination

The proband is a 56-year-old male who was diagnosed with DSN at six years of age following a nerve biopsy (results not available). He had no family history of peripheral neuropathy, and no family consanguinity. His father and mother had normal nerve conduction studies (NCS) in their fifties. His eldest sister also had a normal neurological examination and NCS at age 25, and the proband reported that this sister has not developed symptoms of peripheral neuropathy in her fifties.

At two years of age, his mother noted that he was consistently fatigued and was repeatedly falling when attempting to walk. He also reported loss of sensation in his feet in early childhood and was never able to run. He was not a candidate for ankle-foot orthoses, and subsequently had a triple arthrodesis at age 15 to stabilise his left ankle. He was also diagnosed with scoliosis and had a Harrington rod inserted at age 16 to correct this. During his teenage years, he had multiple unexplained episodes of hemiparesis and double vision after severe migraine pain behind his eyes, and these episodes would last for approximately two weeks. He reports his mother and sister also suffered from frequent severe migraines, particularly between the ages of 30–45.

On examination at the age of 31, he presented with muscle wasting of the hands and feet, as well as pes cavus, hammer toes, corns, and callouses. He walked with a high steppage gait. He had mildly weak hip flexion, and mildly weak ankle dorsiflexion (3/5). He had absent reflexes in the knees and ankles, with distal loss of vibration sense to the knees. There was loss of pain sensation to above the ankles and decreased joint position sense in the toes. He had a tremor of the hands, with weakness of wrist and finger extension. NCS conducted when the patient was 36 years old (Table 1) showed a severe demyelinating motor and sensory neuropathy.

**Table 1:** Nerve conduction study conducted demonstrated a severe motor and sensory demyelinating neuropathy.

	Nerve	Stimulation site	Recording site	Latency (m/s)	Amplitude (mV)
Motor	R. median	Wrist	APB	29.2 (N < 6.2)	1.2 (N > 3)
	R. common peroneal	Ankle	EDB	No response	
	R. tibial	Ankle	AH	21.9 (N < 4.6)	0.5 (N > 3)
		POP FOS	AH	No response	
Sensory	R. sural	Calf	Ankle	No response	
	R. median	Digit 2	Wrist	No response	

Normal values are indicated in brackets next to the value obtained.

APB: abductor pollicis brevis; AH: abductor hallucis; POP FOS: popliteal fossa.

Autonomic testing conducted at this time showed no postural hypotension, normal cardiovascular reflexes, and normal sudomotor reflexes in both upper and lower limbs indicating no significant autonomic neuropathy. An audiogram showed mild bilateral hearing loss above 1500 Hz, and he also reported occasional tinnitus. Right and left ear brainstem auditory evoked potential (BAEP) waveforms were poorly formed with a bilateral delay in wave V. Right and left full and half field visual evoked potentials (VEP) showed delayed P100 waveforms. An MRI of the brain showed multiple subcortical and periventricular white matter hyperintensities on T2 and FLAIR sequences.

**Figure 1:**
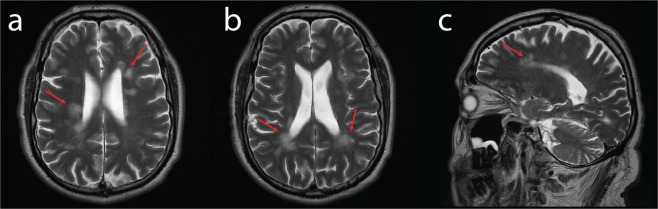
Axial (**a**,**b**) and Sagittal (**c**) T2 weighted images demonstrated multiple white matter hyperintensities, predominantly periventricular in location (red arrows). There were no contrast enhancing lesions suggesting active disease.

On re-examination at age 52, NCS of the left median sensory, left median motor, and left peroneal motor nerves showed no response. He also reported difficulty opposing his thumb and fingers, and a progressive loss of sensation in his right hand. On examination, there was slight curling of the fingers and wasting of the intrinsic hand muscle. Dorsiflexion in his fingers was mildly weak (grade 3/5), as was his finger abduction (grade 3/5). An additional NCS conducted 12 months later additionally showed no response of the left ulnar sensory nerve, and a severely reduced latency of 19.5 m/s in the left ulnar motor nerve. Four years later at the age of 56, his ability to oppose his thumb and fingers had worsened, and he reported that he was unable to write, or use a knife and fork. He additionally had allodynia below his knees. Another brain MRI was conducted at this time (Fig. 1), which again showed multiple bilateral inactive demyelination foci of T2/FLAIR white matter intensity which were mostly periventricular, however they were also present subcortically (mostly in the frontal lobes), at the callososeptal interface and in the right thalamus. There was no restricted diffusion or suspicious enhancement, and the findings were similar to those from an MRI performed age 37 years. A lumbar puncture for oligoclonal bands was not performed.

### Whole-exome sequencing reveals a *de novo EGR2* c. 1232A > G missense variant

Microsatellite marker analysis confirmed the parental paternity of both the proband and his sister (Supplementary Fig. 1). Testing for the *PMP22* duplication and variants in *GJB1* and *MPZ* were both negative. Pathogenic expansions related to SCA1 (*ATXN1*), SCA2 (*ATXN2*), SCA3 (*ATXN3*), SCA6 (*CACNA1A*), SCA7 (*ATXN7*), SCA12 (*PPP2R2B*), and SCA17 (*TBP*) were also excluded.

**Figure 2:**
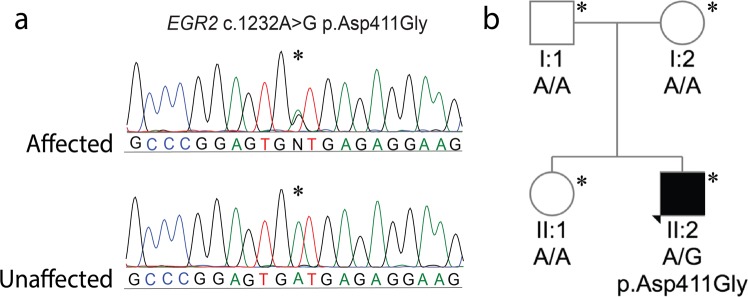
(**a**) Sequencing traces of the variant c.1232A > G *EGR2* in the affected proband (II:2) and the wild-type sequence in the unaffected father (I:1). Genbank sequence NM_000399.3 was used as a reference for the *EGR2* coding sequence. (**b**) Pedigree of the two-generation kindred and associated *EGR2* c.1232A > G genotypes. Solid square denotes the affected male, an open square denotes an unaffected male, and an open circle denotes an unaffected female. An asterisk indicates individuals sent for Sanger sequencing of the *EGR2* c.1232A > G variant.

Whole-exome sequencing (WES) was performed on the proband (II:2). Variant filtering of WES was performed as previously described^19^ and four non-synonymous variants were identified in genes associated with inherited peripheral neuropathy. One variant in the *EGR2* gene (NM_000399.5:c.1232A > G) was not previously reported in variant databases including NCBI dbSNP^20^, 1000 Genomes^21^, gnomAD^22^, and ExAC^23^. Three additional variants were also considered given their low minor allele frequency (MAF <0.1%) (*CACNA1A*, c.6692G > A, NM_023035.2; *BAG3* c.494C > T, NM_004281.3; and *ITPR1* c.6692A > G, NM_001168272.1*)*. Sanger sequencing confirmed the heterozygous *EGR2* c.1232A > G variant in the proband (Fig. 2a), which was absent in the unaffected mother, father, and sister (Fig. 2b). The variants *CACNA1A* c.6692G > A (NM_023035.2), *BAG3* c.494C > T (NM_004281.3), and *ITPR1* c.6692A > G (NM_001168272.1) did not segregate with the peripheral neuropathy phenotype. These results confirmed that the proband is heterozygous for a previously unreported *de novo* variant in the third zinc-finger domain of EGR2 [chr10: 62,813,406T > C (hg38)], leading to the amino acid substitution p.Asp411Gly.

Due to the additional phenotype of hemiplegic migraine in our proband, as well as migraine in his mother and eldest sister, variant filtering of WES was also conducted for genes related to familial hemiplegic migraine (FMH). As variants in *CACNA1A* can also cause an FMH phenotype in addition to an inherited neuropathy, segregation of the *CACNA1A* c.6692G > A variant was conducted. However, it was present in the mother and absent in the FMH-affected eldest sister. Additionally, the proband had no variants in the *ATP1A2* or *SCN1A* genes, which are both known to cause FMH.

### *In silico* analyses show that p.Asp411Gly EGR2 affects a highly conserved residue and that it is likely pathogenic

**Figure 3:**
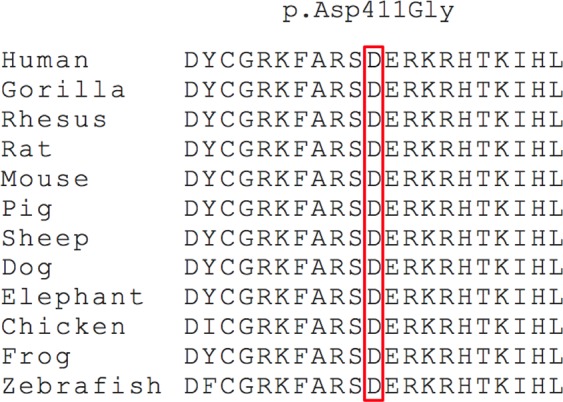
Alignment analysis of the p.Asp411Gly mutation for EGR2 orthologues in different vertebrate species. Position 411 is indicated by the red box.

Amino acid sequence alignment demonstrates that the p.Asp411Gly variant occurs at a highly conserved residue in EGR2 with surrounding amino-acid residues being conserved between orthologues in different vertebrate species (Fig. 3). Multiple *in silico* analysis techniques were utilised to predict the conservation of the Asp411 amino-acid residue and pathogenicity of the p.Asp411Gly variant. GERP^24^, phastCons^25^, and PhyloP^26^ scores all provided further support that this amino-acid residue is highly evolutionarily constrained (Table 2). The functional effect of the p.Asp411Gly variant was predicted to be damaging by Polyphen2^27^, PROVEAN^28^, SIFT^29^, and MutationTaster2^30^ (Table 2).

**Table 2:** Multiple *in silico* analyses support the pathogenicity of the EGR2 p.Asp411Gly variant.

Method	Score	Prediction
GERP	4.85	Highly conserved^a^
phastCons	1	Conserved^b^
PhyloP	4.573	Conserved^c^
Polyphen2 (Human Var)	0.992	Probably damaging^d^
Provean	−3.58	Deleterious^e^
SIFT	0.000	Damaging^f^
MutationAssessor	0.99	Low impact^g^
MutationTaster2	—	Disease causing^h^

^a^GERP scores above + 2 are considered evolutionarily constrained (−11 to + 6). ^b^phastCons scores approaching 1 are predicted to be highly conserved (0.00 to 1.00). ^c^phyloP values that are positive are predicted to be evolutionarily conserved (−14 to + 6). ^d^Polyphen2 scores closer to 1 are predicted to be damaging to protein function (0.00, benign to 0.999, damaging). ^e^Provean scores describe the effect of the protein variation as deleterious or neutral (−13 to 4). ^f^SIFT scores predict the functional impact of a variant (0.00, tolerated to 1.00, damaging). ^g^MutationAssessor describes the predicted functional impact of variant (predicted functional: high or medium to predicted non-functional: low or neutral). ^h^MutationTaster2 predicts the functional impact of a variant: disease causing (probably deleterious), disease causing automatic (previously reported as deleterious), polymorphism (predicted to be non-pathogenic), polymorphism automatic (previously reported as non-pathogenic).

### p.Asp411Gly EGR2 has reduced transcriptional regulatory activity compared to wild-type EGR2 in cultured schwann cells

To determine the relevant functional consequences of the p.Asp411Gly EGR2 variant, the ability of wild-type and p.Asp411Gly EGR2 to induce activity of a previously reported EGR2 response element at the *PMP22* locus was tested in a Schwann cell line^31^. A construct expressing wild-type or p.Asp411Gly EGR2 was transfected into cultured rat Schwann (S16) cells along with a construct harbouring an EGR2 response element upstream of a minimal promoter^32^ and a firefly luciferase (Fluc) reporter gene. To control for cell viability and transfection efficiency, a pCMV-Renilla luciferase (Rluc) construct was also transfected. The fold induction of Fluc, measured as the ratio of Fluc activity to Rluc activity, was employed to test the ability of EGR2 (wild-type or p.Asp411Gly) to activate the *PMP22* enhancer.

**Figure 4:**
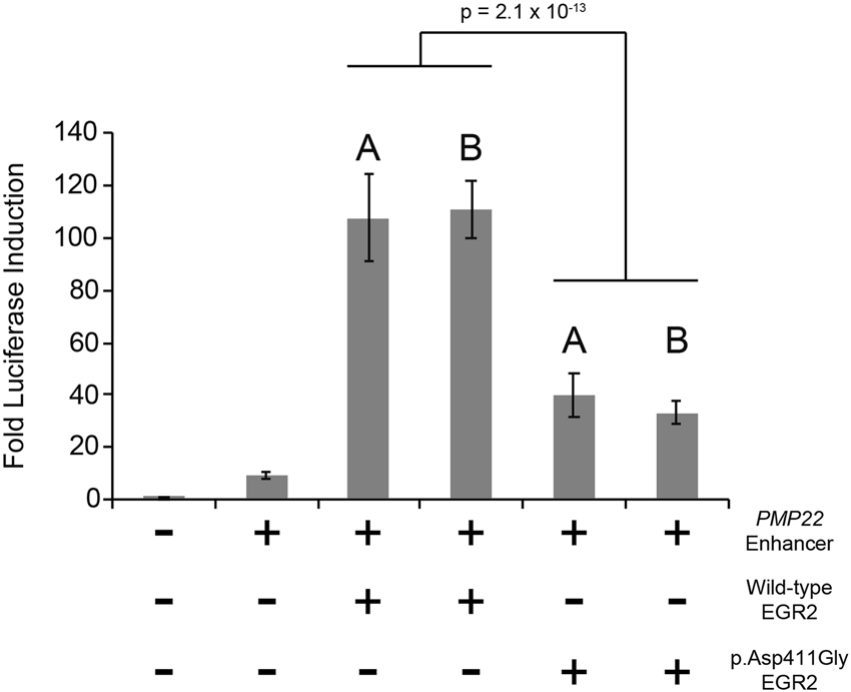
The p.Asp411Gly EGR2 variant decreases transcriptional activation. Wild-type and p.Asp411Gly EGR2 were evaluated for regulatory activity on a previously reported EGR2 response element at the *PMP22* locus. Constructs (conditions indicated along the bottom) were transfected into cultured Schwann (S16) cells and tested for activity in luciferase assays normalized to an empty vector containing no genomic insert (far left column). Wild-type EGR2 and p.Asp411Gly EGR2 were each tested using two independently generated expression constructs (A and B) and each condition was tested in at least 24 biological replicates. The fold induction of Fluc activity is indicated along the y-axis and error bars indicate standard deviations. Statistical significance (p-value) was assessed using a two-tailed Student’s T-test.

These assays demonstrated that wild-type EGR2 was able to increase expression of Fluc (~110 fold increase over the empty vector; Fig. 4) consistent with previous reports that the *PMP22* enhancer includes an EGR2 response element. In contrast, p.Asp411Gly EGR2 induced Fluc activity at a significantly lower level (~40 fold increase over the empty vector p = 2.1 × 10^−13^ compared to wild-type; Fig. 4) indicating that the p.Asp411Gly variant reduces the capacity for EGR2 to appropriately activate the *PMP22* enhancer. These results reveal a functional effect of p.Asp411Gly EGR2 and support the pathogenicity of this variant.

## Discussion

The spectrum of peripheral neuropathy associated with disease-causing *EGR2* variants ranges from severe early onset DSN and CHN, to later onset demyelinating CMT1 and axonal CMT2. Using WES and functional testing we have determined that a *de novo* missense *EGR2* variant, c.1232A > G p.Asp411Gly, causes a severe and early onset DSN phenotype by reducing the capacity for EGR2 to function as a transcription factor. This variant can be classified as ‘pathogenic’ according to guidelines determined by the American College of Medical Genetics and Genomics (PS2, PS3, PM1, PM2, PP2, PP3)^33^. This pathogenic variant affects the third zinc-finger domain of EGR2, which is highly conserved among species and is a DNA-binding motif crucial for its role as a transcription factor in the regulation of myelin genes^34^. All reported pathogenic variants are within these three zinc-finger regions except for p.Ile268Asn, which causes recessive CHN and is within an inhibitory domain (Table 3)^4^.

**Table 3:** Pathogenic *EGR2* variants are clinically heterogeneous, and there is no apparent association between the affected EGR2 domain and the resultant phenotype.

Domain	Variant	Phenotype	Reference
NAB repressor binding site	p.Ile268Asn	CHN	Warner *et al*., 1998^4^
Zinc-finger 1	p.Arg353Gly	CMT	Nakamura *et al*., 2012^48^
	p.Asp355Val	CMT	Bellone *et al*., 1999^6^
	p.Asp355Gly	CMT	Wang *et al*., 2016^49^
	p.Arg359Gln	CMT	Mikešová *et al*., 2001^9^
	p.Arg359Trp	DSN	Timmerman *et al*., 1999^1^
		CHN	Boerkoel *et al*., 2001^5^
		DSN	Chung *et al*., 2005^3^
		DSN	Gargaun *et al*., 2016^36^
Zinc-finger 2	p.Arg381Cys	CMT	Yoshihara *et al*., 2001^8^
		CMT	Briani *et al*., 2010^11^
		CMT	Wang *et al*., 2016^49^
	p.Arg381Leu	CMT	Fusco *et al*., 2019^50^
	p.Arg381His	CMT	Pareyson *et al*., 2000^7^
	p.Asp383Tyr	DSN	Numakura *et al*., 2003^2^
	p.Ser382Arg + p.Asp383Tyr	CHN	Warner *et al*., 1998^4^
	p.Thr387Asn	CMT	Shiga *et al*., 2012^10^
Zinc-finger 3	p.Arg409Trp	CMT	Warner *et al*., 1998^4^
		CMT	Leonardi *et al*., 2019
	p.Arg409Gln	CMT	Sevilla *et al*., 2015^13^
	p.Asp411Gly	DSN	This study
	p.Glu412Gly	CMT	Safka Brožková *et al*., 2012^12^
		CMT	Tozza *et al*., 2019^14^
	p.Glu412Lys	DSN	Szigeti *et al*., 2007^35^

Functional testing conducted here with luciferase-based reporter assays demonstrated that p.Asp411Gly EGR2 activates transcription less effectively than wild-type EGR2, similar to what has been previously reported for pathogenic *EGR2* variants located in the zinc-finger domains^13,15,34,35^. EGR2 function is crucial for myelination of the peripheral nervous system, and *Krox20*^−/−^ (murine *EGR2* orthologue) mice demonstrated arrested differentiation of Schwann cells at an early stage^17^. Interestingly, heterozygous *Krox20*^+/−^ mice are phenotypically normal^17^, yet all reported pathogenic *EGR2* variants in the zinc-finger domains cause peripheral neuropathy in a heterozygous state (Table 3)^13^. This is likely explained by the finding that in addition to pathogenic *EGR2* variants resulting in a decrease or loss of EGR2 function, it has been shown that pathogenic *EGR2* variants in all three zinc-finger domains (p.Arg359Trp, and p.Ser382Arg + p.Asp383Tyr, and p.Arg409Trp) result in the dominant-negative inhibition of wild-type EGR2^15,18^ and SOX10^18^. These results are consistent with the reduction in transcriptional activation we observed in our luciferase assays with p.Asp411Gly EGR2.

The severity and onset of peripheral neuropathy caused by *EGR2* variants is highly heterogenous, and it is yet to be determined why this broad phenotypic variability exists. This variation is exemplified by the p.Arg359Trp variant, which causes demyelinating CMT1D^3^, DSN^5,36^, and CHN^5^. This is also true for different variants of the amino acid adjacent to the p.Asp411Gly variant we have reported here, where p.Glu412Gly causes both demyelinating CMT1D^12^ and axonal CMT^14^, and p.Glu412Lys causes DSN^35^. Given these observations, it is possible that additional genetic factors exist which modify the phenotype of *EGR2* variant, such as those recently reported for CMT1A^37^.

Our patient had findings on brain MRI and evoked potentials that may be consistent with multiple sclerosis, however it was determined that he did not fulfil diagnostic criteria. White matter lesions have been reported in some patients with CMT^38–41^, and the proband had no contrast enhancing lesions suggesting active demyelinating disease in both brain MRIs conducted. In CMTX1, which is caused by pathogenic variants in *GJB1*, white matter lesions were reported in 7.5% of a large cohort^42^. Given that EGR2 mediates transcription of *GJB1*, it is possible that the reduced efficiency of p.Asp411Gly EGR2 in activating transcription resulting in a phenotype similar to that seen in some CMTX1 patients. Additionally, the FMH phenotype may be a chance association, or due to another genetic cause that has not been identified, as there were no variants in hemiplegic migraine genes that segregated with the phenotype. White matter lesions have also been reported in patients with migraines and patients with sporadic hemiplegic migraine^43–46^, further complicating the phenotype seen in our proband.

In this study we report a novel *de novo EGR2* variant, c.1232A > G p.Asp411Gly, as the cause of severe, early-onset CMT3 (DSN). We provide robust functional data to support the deleterious functional effect of this mutation. The phenotype is complicated by clinical features of white matter lesions and FMH which may be a chance association, although it would be important to consider the possibility of central nervous system involvement in future cases of EGR2-related neuropathies.

## Methods

### Subjects

The proband and three family members were recruited and informed consent was obtained for this study using protocols approved by the Sydney Local Health District Human Ethics Research Committee (SLHD HERC). All experiments were performed in accordance with relevant guidelines and regulations from SLHD HREC. Genomic DNA was extracted from peripheral blood using the PureGene Kit (Qiagen) following manufacturer’s instructions.

### Whole-exome sequencing (WES)

WES was performed on genomic DNA (2.5 µg) in the proband and was outsourced to Macrogen (South Korea) as previously described^19^.

### Bioinformatic analysis

Evolutionary conservation of the EGR2 D11 amino acid reside was conducted using GERP (http://mendel.stanford.edu/sidowlab/downloads/gerp/index.html), phastCons and PhyloP (http://compgen.cshl.edu/phastweb/runtool.php). The predicted functional effect of the EGR2 p.Asp411Gly mutation was assessed using Polyphen2 (http://genetics.bwh.harvard.edu/pph2/), PROVEAN (http://provean.jcvi.org/seq_submit.php), SIFT (https://sift.bii.a-star.edu.sg/www/SIFT_seq_submit2.html), MutationAssessor (http://mutationassessor.org/r3/), and MutationTaster2 (http://www.mutationtaster.org/).

### Sanger sequencing

All primers and PCR conditions are available upon request. PCR amplicons were sent to Garvan Molecular Genetics, Garvan Institute (Sydney, Australia) for Sanger sequencing using BigDye Terminator cycle sequencing protocols and visualised using Sequencher 2.3 software (Gene Codes Corporation).

### Haplotype analysis

PCR amplicons from microsatellite markers (D1S347, D1S249, D2S2228, D2S140, D16S519) were sent for fragment analysis at Garvan Molecular Genetics, Garvan Institute (Sydney, Australia), and visualised using GeneMarker (SoftGenetics). Microsatellite marker size was determined using GS600 size standard.

### Luciferase reporter gene expression constructs

Mutagenic primers were designed to model the p.Asp411Gly *EGR2* mutation in an expression construct containing the mouse *EGR2* open-reading frame directed by the CMV promoter (gift of John Svaren, University of Wisconsin). Site-directed mutagenesis was performed using the QuikChange II XL Site-Directed Mutagenesis Kit (Agilent Technologies) and the manufacturer’s instructions. Mutant constructs were verified by Sanger sequencing (University of Michigan Medical School DNA Sequencing Core).

### Cell culture, transfection, and luciferase assays

Cultured rat Schwann cells (S16)^47^ were grown in Dulbecco’s Modified Eagle’s Medium (DMEM) with 10% (v/v) fetal bovine serum (Gibco), 2 mM L-glutamine (Gibco), 50 U/mL penicillin, and 50 g/mL streptomycin. For luciferase assays, ~1 × 10^4^ S16 cells were plated in each well of a 96-well untreated cell culture plate and grown overnight at 37 °C with 5% CO_2_. After 24 h, the cells were transfected with the experimental constructs using Lipofectamine 2000 (Invitrogen) diluted 1:100 in OptiMem (Life Technologies). Cells in each well received 200 ng of an expression construct: either empty pEIB (expression vector containing a Fluc reporter gene with no genomic insert)^32^ or the pEIB-Forward vector with a *PMP22* enhancer^31^ upstream of a Fluc reporter gene. S16 cells were co-transfected with 100 ng per well of wild-type or mutant *EGR2* constructs in addition to the *PMP22* enhancer construct. For an internal control for transfection efficiency, 2 ng of a pCMV-Rluc construct were transfected into cells in all wells. Each experimental construct was diluted in OptiMem and incubated with an equal volume of Lipofectamine 2000 in OptiMem for 20 min at room temperature before being applied to cells. Cells were incubated with transfection reagents for 4 h at 37 °C with 5% CO_2_, at which point the transfection reagents were removed and replaced with standard growth medium (above) and allowed to grow for 48 h at 37 °C with 5% CO_2_.

After 48 h, growth medium was removed, and cells were washed with 1X PBS. The cells were then lysed for 1 h at room temperature in 1X Passive Lysis Buffer (Promega), and 10 uL of lysate from each well was transferred into a white polystyrene 96-well plate (Corning). A Dual Luciferase Assay was performed using a Glomax Multi-Detection System and a Dual Luciferase Reporter 1000 Assay System kit (Promega) to determine Fluc and Rluc activities.

The ratio of Fluc to RLuc activity in each well was calculated, and ratios from individual wells were normalized relative to the average Fluc to Rluc ratio from the empty pEIB vector. The mean normalized ratio is a readout for the fold induction of Fluc by each experimental construct, and this is shown with standard deviation in the figure. Each variant was tested with two independently generated constructs (A and B in the figure) to rule out expression changes due to variation in the expression construct backbone, and each construct was tested in at least 24 individual experiments. Statistical significance was assessed using a two-tailed Student’s T-test.

## Supplementary information

Supplementary Information

## Data Availability

The datasets generated during and/or analysed during the current study are available from the corresponding author on reasonable request.
