# Supplementary material for: A de novo EGR2 variant, c.1232A > G p.Asp411Gly, causes severe early-onset Charcot-Marie-Tooth Neuropathy Type 3 (Dejerine-Sottas Neuropathy)
Source: Sci Rep. 2019 Dec 18;9:19336. doi: 10.1038/s41598-019-55875-4 (PMC6920433; doi:10.1038/s41598-019-55875-4)
Supplement: Supplementary file 1 — Supplementary Information [file 41598_2019_55875_MOESM1_ESM.pdf]

A *de novo EGR2* variant, c.1232A>G p.Asp411Gly, causes severe early-onset Charcot-Marie-Tooth Neuropathy Type 3 (Dejerine-Sottas Neuropathy)

Bianca R. Grosz[1,2*], Natasha B. Golovchenko[3], Melina Ellis[1], Kishore Kumar[2,4,5], Garth A. Nicholson[1,2,4,5], Anthony Antonellis[3,6], Marina L. Kennerson[1,2,4*]

1. Northcott Neuroscience Laboratory, ANZAC Research Institute, NSW, Australia.
2. Sydney Medical School, University of Sydney, NSW, Australia.
3. Department of Human Genetics, University of Michigan Medical School, Ann Arbor, MI USA
4. Molecular Medicine Laboratory, Concord Repatriation General Hospital, NSW, Australia.
5. Department of Neurology, Concord Repatriation General Hospital, NSW, Australia
6. Department of Neurology, University of Michigan Medical School, Ann Arbor, MI USA

Supplementary Data

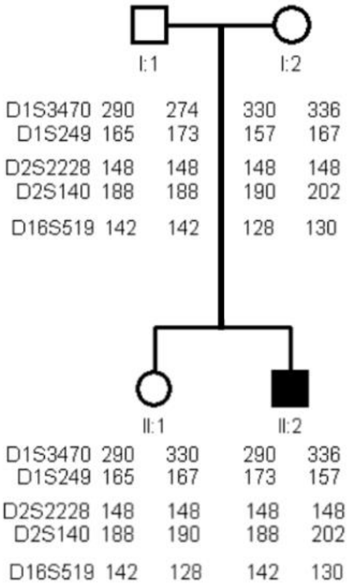

S1: Haplotype analysis of five microsatellite markers confirms paternity of the proband and the eldest sister.
